# Significant changes in advanced lung cancer survival during the past decade in Hungary: impact of modern immunotherapy and the COVID-19 pandemic

**DOI:** 10.3389/fonc.2023.1207295

**Published:** 2023-10-04

**Authors:** Zoltán Kiss, Gabriella Gálffy, Veronika Müller, Judit Moldvay, Veronika Sárosi, Zsolt Pápai-Székely, Edit Csada, Anna Kerpel-Fronius, Zsolt Király, Zoltán Szász, Gábor Hódi, Zoltán Polányi, Krisztina Kovács, Eugenia Karamousouli, Kata Knollmajer, Tamás G. Szabó, Andrea Berta, Zoltán Vokó, György Rokszin, Zsolt Abonyi-Tóth, Zsófia Barcza, Lilla Tamási, Krisztina Bogos

**Affiliations:** ^1^ MSD Pharma Hungary Ltd, Budapest, Hungary; ^2^ Second Department of Medicine and Nephrology-Diabetes Center, University of Pécs Medical School, Pécs, Hungary; ^3^ Pulmonology Hospital Törökbálint, Department of Pulmonology, Törökbálin, Hungary; ^4^ Department of Pulmonology, Semmelweis University, Budapest, Hungary; ^5^ 1st Department of Pulmonology, National Korányi Institute of Pulmonology, Budapest, Hungary; ^6^ 2nd Department of Pathology, MTA-SE NAP, Brain Metastasis Research Group, Hungarian Academy of Sciences, Semmelweis University, Budapest, Hungary; ^7^ Faculty of Medicine, University of Pécs, Pécs, Hungary; ^8^ Fejér County Szent György, University Teaching Hospital, Székesfehérvár, Hungary; ^9^ Csongrád-Csanád County Hospital for Chest Diseases, Deszk, Hungary; ^10^ National Korányi Institute of Pulmonology, Department of Radiology, Budapest, Hungary; ^11^ Veszprém County Pulmonary Hospital, Farkasgyepű, Hungary; ^12^ Department of Pulmonology, Petz Aladár University Teaching Hospital, Győr, Hungary; ^13^ MSD, Athens, Greece; ^14^ Center for Health Technology Assessment, Semmelweis University, Budapest, Hungary; ^15^ Syreon Research Institute, Budapest, Hungary; ^16^ RxTarget Ltd., Szolnok, Hungary; ^17^ Department of Biostatistics, University of Veterinary Medicine, Budapest, Hungary; ^18^ Syntesia Ltd, Budapest, Hungary; ^19^ National Korányi Institute of Pulmonology, Budapest, Hungary

**Keywords:** lung cancer, long-term survival, mortality, Hungary, NSCLC, adenocarcinoma lung, squamous cell carcinoma lung

## Abstract

**Objective:**

The approval of immunotherapy (I-O) for the treatment of late-stage non-small cell lung cancer (NSCLC) opened new perspectives in improving survival outcomes. However, survival data have not yet been provided from the period of the Covid-19 pandemic. The aims of our study were to assess and compare survival outcomes of patients with advanced LC receiving systemic anticancer treatment (SACT) before and after the approval of immunotherapy in Hungary, and to examine the impact of pandemic on survival outcomes using data from the Hungarian National Health Insurance Fund (NHIF) database.

**Methods:**

This retrospective, longitudinal study included patients aged ≥20 years who were diagnosed with advanced stage lung cancer (LC) (ICD-10 C34) between 1 January 2011 and 31 December 2021 and received SACT treatment without LC-related surgery. Survival rates were evaluated by year of diagnosis, sex, age, and LC histology.

**Results:**

In total, 35,416 patients were newly diagnosed with advanced LC and received SACT during the study period (mean age at diagnosis: 62.1–66.3 years). In patients with non-squamous cell carcinoma, 3-year survival was significantly higher among those diagnosed in 2019 vs. 2011–2012 (28.7% [95% CI: 26.4%–30.9%] vs. 14.45% [95% CI: 13.21%–15.69%], respectively). In patients with squamous cell carcinoma, 3-year survival rates were 22.3% (95% CI: 19.4%–25.2%) and 13.37% (95% CI: 11.8%–15.0%) in 2019 and 2011–2012, respectively, the change was statistically significant. Compared to 2011–2012, the hazard ratio of survival change for non-squamous cell carcinoma patients was 0.91, 0.82, and 0.62 in 2015–2016, 2017–2018, and 2019, respectively (p<0.001 for all cases). In the squamous cell carcinoma group, corresponding hazard ratios were 0.93, 0.87, and 0.78, respectively (p<0.001 for all cases). Survival improvements remained significant in both patient populations during the Covid-19 pandemic (2020–2021). No significant improvements were found in the survival of patients with small cell carcinoma. Platinum-based chemotherapy was the most common first-line treatment in all diagnostic periods, however, the proportion of patients receiving first- or second-line immunotherapy significantly increased during the study period.

**Conclusion:**

3-year survival rates of NSCLC almost doubled among patients with non-squamous cell carcinoma and significantly improved at squamous cell carcinoma over the past decade in Hungary. Improvements could potentially be attributable by the introduction of immunotherapy and were not offset by the Covid-19 pandemic.

## Introduction

Lung cancer (LC) is a rapidly progressing, life-threatening disease which constitutes a major disease burden (1–3). LC still has one of the lowest survival rates among carcinomas, making it one of the most important health problems of the 21^st^ century (4, 5). Before the development of immunotherapy (I-O), advanced non-small cell LC (NSCLC) had poor outcomes, with only 15%–25% of patients surviving the end of the second year and very few still alive at 5 years after having received systemic treatment (6–8). In a Canadian study conducted between 2010–2015, 5-year survival rates of stage IV NSCLC were 5.9% with squamous histology, 5.2% with non-squamous histology and chemotherapy, and 12.9% with non-squamous histology and targeted therapy (9). A real-world study from Portugal reported a 2-year survival rate of 13% in patients with stage IV NSCLC, and nearly a quarter of patients did not receive SACT (6).

The development and subsequent approval of immunotherapy drugs for NSCLC including the immune checkpoint inhibitors pembrolizumab, atezolizumab, and nivolumab brought about significant improvements in the short- and long-term prognosis of LC either in combination with chemotherapy or as monotherapy. In the pivotal KEYNOTE studies, pembrolizumab in mono or in combination therapy provided significant overall survival (OS) benefits for patients with advanced NSCLC compared to docetaxel and platinum-based chemotherapy, regardless of programmed death-ligand 1 (PD-L1) tumor proportion score (TPS) (10–13). Atezolizumab and nivolumab were also associated with significant improvements in long-term OS compared to docetaxel among patients with previously treated, advanced NSCLC in their respective clinical trials (14–16).

A growing number of real-world studies have confirmed the survival improvement associated with immunotherapy in patients with NSCLC, comparing outcomes in the pre- and post-immunotherapy era (17, 18). However, certain studies suggest an efficacy–effectiveness gap between clinical trials and real-world settings, suggesting that there is still room for improvement in terms of maximizing the benefits of immunotherapy in routine clinical practice (17, 19). Furthermore, patient populations included in these studies were different from clinical study populations.

The Covid-19 pandemic led to significant disruptions in all aspects of healthcare including cancer screening, diagnosis, and treatment, especially during the first waves (20, 21). Patients with LC are at a particularly high risk of Covid-19 related morbidity and mortality, and the presence of LC significantly increases the risk of mortality from SARS-CoV-2 infection (22–24). Accordingly, studies investigating the relationship between Covid-19 and LC have reported higher Covid-19 related mortality rates in LC patients compared to the general population, and an increase in LC mortality during the pandemic compared to the preceding years (25, 26).

So far, no comparative data have been published on the survival outcomes of LC patients before and after the introduction of immunotherapy in Hungary. Therefore, the main goal of our study (called HeLP3.2 study) was to examine the OS of patients with advanced LC receiving systemic anticancer treatment (SACT) in the pre- and post-immunotherapy eras. Furthermore, we aimed to compare survival rates according to age, sex, and LC histology (squamous cell carcinoma, non-squamous cell carcinoma, small cell carcinoma). Finally, we sought to investigate the impact of the Covid-19 pandemic on the OS of patients with advanced LC.

## Materials and methods

### Study design

The nationwide, retrospective HeLP3.2 study (Hungarian Evaluation of Lung Cancer Patient Pathway) was based on the claims database of the National Health Insurance Fund of Hungary (NHIF) which is a nationwide insurance system covering almost 100% of the Hungarian population. The NHIF database contains medical information regarding ID and ICD-10 codes of inpatient admissions and out-patient visits and procedures, containing 100% of LC-related interventions as there is no other insurance system covering LC treatment in Hungary. The study was approved by the National Ethical Committee (ethical approval number IV/3940- 3/2021/EKU).

The HeLP3.2 study included patients newly diagnosed with LC (ICD-10 C34) between 1 January 2011 and 31 December 2021 who were ≥20 years old at the time of diagnosis. A reference screening period was set for 2009–2010 to identify newly diagnosed LC patients from 2011. The potential miscoding of LC was avoided by only including patients with a minimum of two records of the C34 ICD-10 code within an interval of over 30 but less than 365 days following the first coding. Patients with only one recorded C34 code who died within 60 days after coding were also included. Patients with ICD-10 codes related to other cancers and those receiving oncological treatments other than the LC-specific treatment protocol 6 months prior to or 12 months following the first recorded LC code were excluded from the analysis unless they had an LC-related histology code or LC-related treatment records in the NHIF database.

For the current analyses, we only included LC patients who received first-line SACT without undergoing surgery during the first 180 days after diagnosis. SACT included chemotherapy, targeted therapy, or immunotherapy based on special reference codes in NHIF database records. Similar to in the I-O Optimize Initiative studies, our study included patients with advanced LC (27). Staging information was not available for the analysis from the NHIF database, however, the analysis included LC patients who received SACT treatment without LC-related surgery in their records after LC diagnosis. Therefore, based on local and international guidelines, we can assume that the vast majority of patients were in advanced stages (IIIB or IV). LC histology was recorded in the database for almost 95% of the selected patient population, therefore, we conducted separate survival analyses for patients with non-squamous cell carcinoma, squamous cell carcinoma, and small cell carcinoma, only for those who received SACT treatment.

Patients newly diagnosed with LC were followed until 30 June 2022 or alternatively until the time of death based on the NHIF database. Therefore, we could follow the survival of included patients from the time of diagnosis through different lines of therapy, even in the case of remission-relapse phases until the date of death or the end of the study period. We did not exclude those patients, whom had relapse, but followed them till the end of study period or death. The database does not contain cause-specific mortality data; therefore, all-cause mortality was assessed. The NHIF updates the date of death on a monthly basis in accordance with the reports from the State Population Registry Office. Data were anonymized during data collection, and only non-identifiable data were used for analysis.

**Figure 1:**
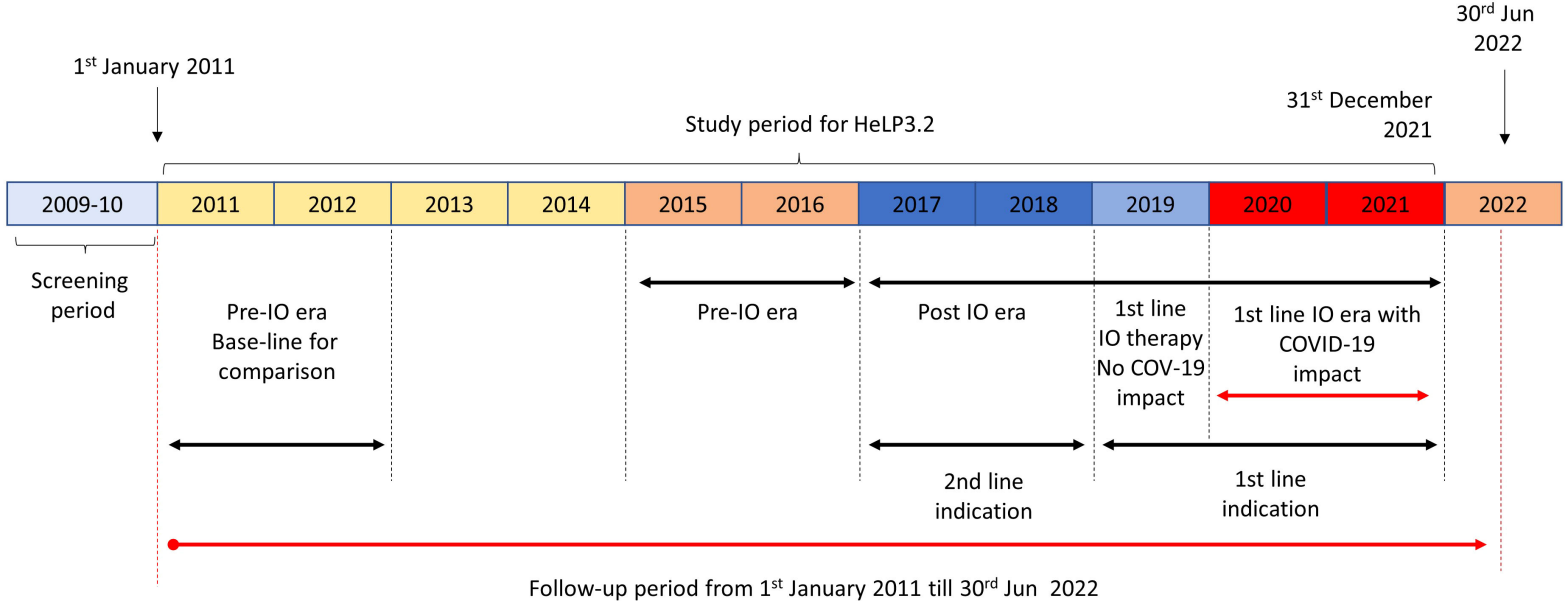
HeLP3.2 study periods: 2011–2012, baseline pre-immunotherapy era for comparisons; 2015–2016, pre-immunotherapy era; 2017–2018, immunotherapy era with second-line indication for immunotherapy in NSCLC; 2019, immunotherapy era with first-line indication for immunotherapy in NSCLC; 2020–2021, Covid-19 pandemic. I-O, immunotherapy; NSCLC, non-small cell lung cancer.

The survival of LC patients after receiving first SACT was compared between the following periods: (i) 2011–2012, serving as a baseline period for comparisons within the pre-immunotherapy era; (ii) 2015–2016, the end of the pre-immunotherapy era; (iii) 2017–2018, representing the availability of second-line immunotherapy in NSCLC; (iv) 2019, representing the first-year of first-line immunotherapy approval, (v) 2020–2021, to evaluate the impact of the Covid-19 pandemic on the survival of advanced LC (**Figure 1**). The selected time periods were based on the dates of public reimbursement approval of first- or second-line immunotherapy in Hungary. The reimbursement status of different modern LC treatments was the following during the study period: pembrolizumab first became available in the form of named-patient basis access for the second-line treatment of adenocarcinoma in 2016 and for squamous cell carcinoma in 2015, it was granted general reimbursement in these indications in 2017–2018 and became reimbursed for the first-line treatment of adenocarcinoma and squamous cell carcinoma in October 2018. Nivolumab received reimbursement in the second-line treatment setting for the treatment of adenocarcinoma and for squamous cell carcinoma in 2015, while atezolizumab gained reimbursement in 2017. Nivolumab and atezolizumab had no first-line indication during the study period. During the analysis of the 3-year survival rate, comparisons were made between the first 3-year survival period of LC patients who were diagnosed in 2011-2012 and those who were diagnosed during the 2017-18 period. It is important to note that in each instance of periods, the comparisons were initiated subsequent to the LC diagnoses.

The total number of newly diagnosed LC patients per year is shown as crude numbers (n). The mean age at diagnosis was determined for all patients and according to sex for each diagnostic period. Overall survival (OS) was calculated and reported as defined by Tan et al. (28) Survival rates were calculated for each type of LC histology where pertinent data were available. Kaplan-Meier curves were drawn to show 3-year and long-term OS. The hazard ratio of death among patients diagnosed in different diagnostic periods versus the baseline period of 2011–2012 was calculated using Cox regression. The effect of age and sex on survival outcomes was examined by subgroup analyses. For a given diagnostic period, 12-, 24-, 36-, 48-, and 60-month survival rates were calculated, where available, and the absolute change in survival at a given follow-up time was also determined according to age at diagnosis and LC histology. The distribution and sequence of different lines of SACT in different study periods were also analyzed according to LC histology (platinum-based therapy, targeted therapy, immunotherapy, non-platinum-based therapy).

All calculations were performed with R version 4.2.1 (13/10/2022) with package survival version 3.4-0.

## Results

### Study population

Between 1 January 2011 and 31 December 2021, 89,866 patients were newly diagnosed with LC based on the NHIF database (total LC patient population) (**Supplementary Table 1**). Of them, 35,416 (39.4%) received first-line SACT without undergoing surgery during the first 180 days after diagnosis, therefore, these patients were considered to have late-stage, advanced LC and were eligible for inclusion in the analyses. The highest proportion of advanced LC patients was observed in 2021 when this patient population accounted for 41.9% of the total LC patient population.

The mean age of the SACT population at diagnosis increased from 62.1 (SD: ± 8.0) years in 2011–2012 to 66.3 (SD ±8.0) in 2021 and was similar in female and male patients throughout the whole study period (mean: 63.5 ± 8.1 in males vs. 63.7 ± 8.0 years in females) (**Table 1**). The highest number of patients was recorded in the age group of 60–69 years (n=16,666, 47.1%); patients aged 20–49 years represented 4.8% of the total advanced LC patient population.

**Table 1:** Main characteristics of patients with advanced LC who were diagnosed between 1 January 2011 and 31 December 2021 and received first-line SACT.

	Number of Patients															
	2011-2012		2013-2014		2015-2016		2017-2018		2019		2020		2021		Total	
Patients with new LC diagnosis (n)	6,507		6,628		6,556		6,642		3,232		2,885		2,966		35,416	
Male (n, % of LC patients)	4,153	63.82%	4,066	61.35%	4,023	61.36%	4,010	60.37%	1,816	56.19%	1,687	58.47%	1,702	57.38%	21,457	60.59%
Female (n, % of LC patients)	2,354	36.18%	2,562	38.65%	2,533	38.64%	2,632	39.63%	1,416	43.81%	1,198	41.53%	1,264	42.62%	13,959	39.41%
Mean age at diagnosis (y, mean ±SD)	62.09	±8.02	62.92	±8.09	63.79	±7.75	64.41	±7.90	65.45	±8.13	65.58	±8.18	66.29	±8.00	63.89	±7.98
Male (y, mean ±SD)	61.70	±8.46	62.24	±8.28	62.92	±8.06	64.12	±7.79	64.93	±7.63	64.98	±8.11	65.81	±8.04	63.46	±8.08
Female (y, mean ±SD)	61.95	±8.19	62.66	±8.17	63.45	±7.88	64.30	±7.86	65.22	±7.92	65.33	±8.16	66.08	±8.02	63.72	±8.03
Age groups																
20-49	421	6.47%	379	5.72%	315	4.80%	264	3.97%	107	3.31%	118	4.09%	92	3.10%	1,696	4.79%
50-59	2,083	32.01%	1,900	28.67%	1,530	23.34%	1,381	20.79%	594	18.38%	504	17.47%	470	15.85%	8,462	23.89%
60-69	2,795	42.95%	2,977	44.92%	3,287	50.14%	3,275	49.31%	1,583	48.98%	1,348	46.72%	1,401	47.24%	16,666	47.06%
70 and over	1,208	18.56%	1,372	20.70%	1,424	21.72%	1,722	25.93%	948	29.33%	915	31.72%	1,003	33.82%	8,592	24.26%
Morphology																
Squamous cell carcinoma	1,765	27.12%	1,768	26.67%	1,772	27.03%	1,754	26.41%	840	25.99%	738	25.58%	768	25.89%	9,405	26.56%
Adenocarcinoma	3,087	47.44%	3,143	47.42%	3,114	47.50%	3,251	48.95%	1,620	50.12%	1,470	50.95%	1,515	51.08%	17,200	48.57%
Small-cell carcinoma	1,304	20.04%	1,426	21.51%	1,346	20.53%	1,333	20.07%	632	19.55%	567	19.65%	549	18.51%	7,157	20.21%
Morphology not specified	351	5.39%	291	4.39%	324	4.94%	304	4.58%	140	4.33%	110	3.81%	134	4.52%	1,654	4.67%
Male	4,153		4,066		4,023		4,010		1,816		1,687		1,702		21,457	
Squamous cell carcinoma	1,333	32.10%	1,297	31.90%	1,290	32.07%	1,259	31.40%	560	30.84%	517	30.65%	541	31.79%	6,797	31.68%
Adenocarcinoma	1,831	44.09%	1,815	44.64%	1,790	44.49%	1,876	46.78%	858	47.25%	807	47.84%	790	46.42%	9,767	45.52%
Small-cell carcinoma	773	18.61%	770	18.94%	725	18.02%	700	17.46%	319	17.57%	306	18.14%	295	17.33%	3,888	18.12%
Morphology not specified	216	5.20%	184	4.53%	218	5.42%	175	4.36%	79	4.35%	57	3.38%	76	4.47%	1,005	4.68%
Female	2,354		2,562		2,533		2,632		1,416		1,198		1,264		13,959	
Squamous cell carcinoma	432	18.35%	471	18.38%	482	19.03%	495	18.81%	280	19.77%	221	18.45%	227	17.96%	2,608	18.68%
Adenocarcinoma	1,256	53.36%	1,328	51.83%	1,324	52.27%	1,375	52.24%	762	53.81%	663	55.34%	725	57.36%	7,433	53.25%
Small-cell carcinoma	531	22.56%	656	25.60%	621	24.52%	633	24.05%	313	22.10%	261	21.79%	254	20.09%	3,269	23.42%
Morphology not specified	135	5.73%	107	4.18%	106	4.18%	129	4.90%	61	4.31%	53	4.42%	58	4.59%	649	4.65%
Mean follow-up (months)																
Squamous cell carcinoma	21.98		Not evaluated		22.00		21.38		20.15		15.63		9.91			
Adenocarcinoma	21.25		Not evaluated		20.67		19.86		18.43		15.36		10.01			
Small-cell carcinoma	17.96		Not evaluated		15.72		15.20		13.68		12.36		8.90			

LC, lung cancer; SACT, systemic anticancer therapy; SD, standard deviation.

Tumor histology was recorded in 95.3% of all cases. The majority of patients had NSCLC (75.1%). Across all LC patients, 48.6% had non-squamous cell carcinoma (NSQ), 26.6% had squamous cell carcinoma, and 20.2% had small cell LC. Non-squamous cell carcinoma was more common among females (53.3% vs. 45.5% in men), while squamous cell carcinoma was more frequent in males (31.7% vs. 18.7% in women).

### Long-term survival

**Figure 2:**
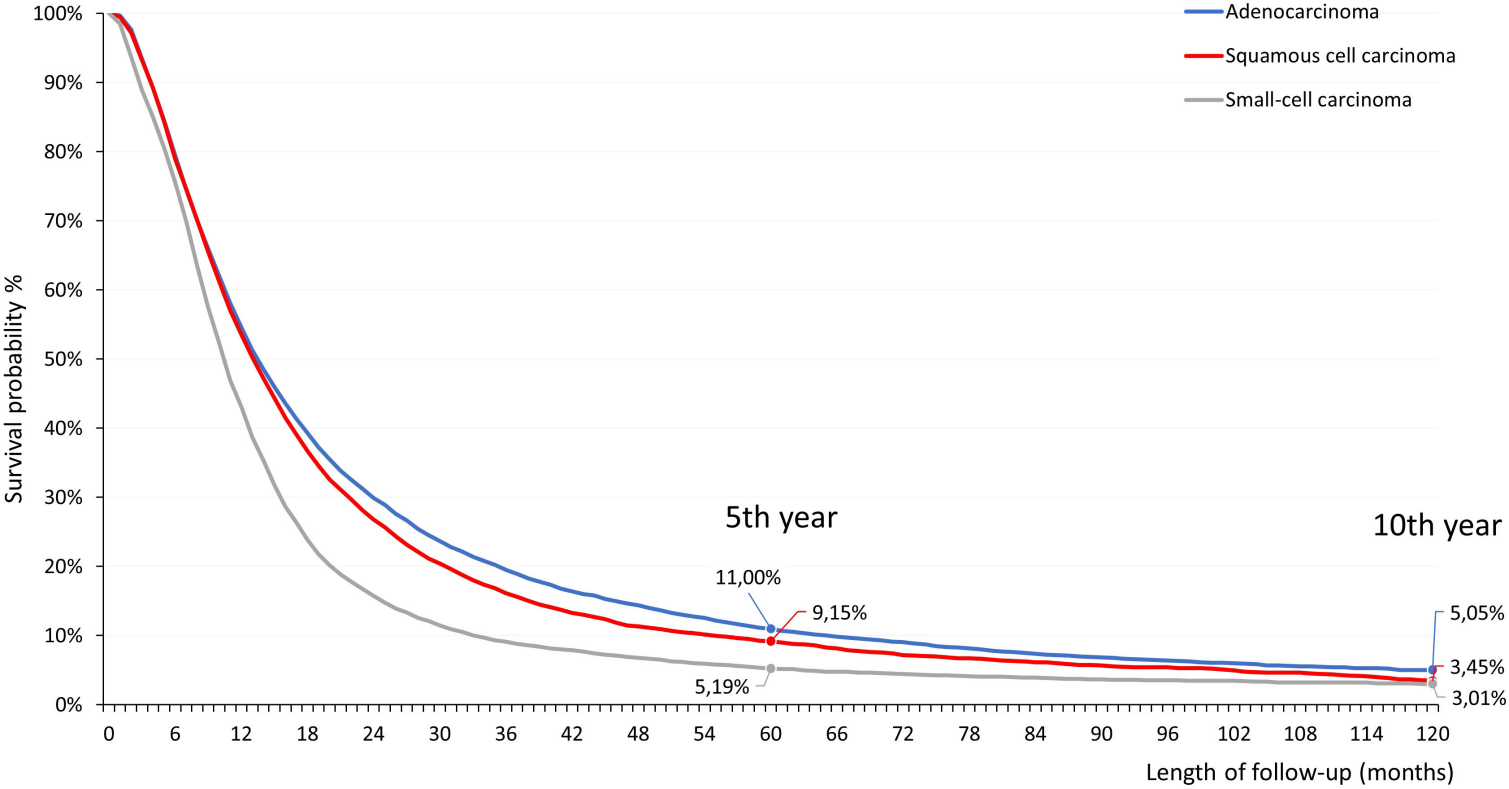
Five- and ten-year estimated OS of patients with advanced LC who were diagnosed between 1 January 2011 and 31 December 2021 and received first-line SACT. LC, lung cancer; OS, overall survival; SACT, systemic anticancer treatment.

Among patients diagnosed in 2011–2021 period, 5-year survival was 11.0% in those with non-squamous cell carcinoma, 9.2% in patients with squamous cell carcinoma, and 5.2% among patients with small cell LC (**Figure 2**).

### Survival estimates for different study periods

**Figure 3:**
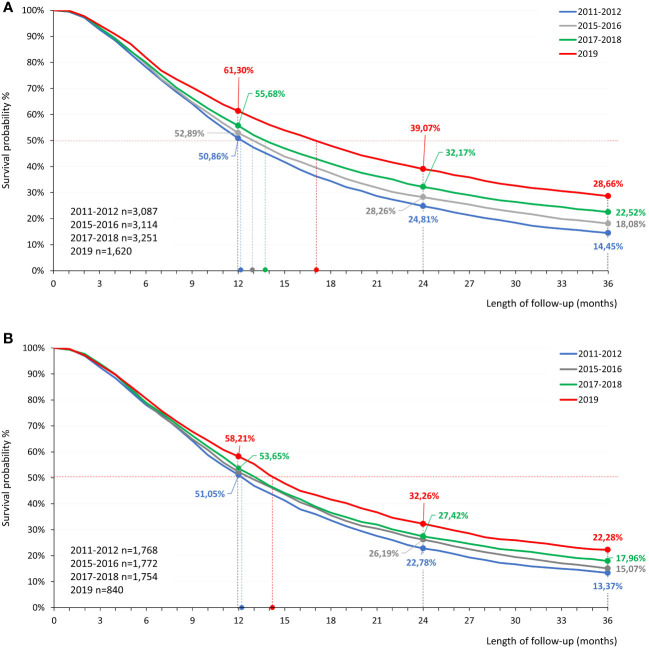
Estimated 3-year OS of patients with advanced non-squamous cell carcinoma **(A)** and squamous cell carcinoma **(B)** who were diagnosed between 2011–2012, 2015–2016, 2017–2018, and 2019 and received SACT. LC, lung cancer; OS, overall survival; SACT, systemic anticancer therapy.

**Figure 3** shows 3-year survival among patients with non-squamous cell carcinoma (**Figure 3A**) and squamous cell carcinoma (**Figure 3B**) diagnosed in different study periods. During the baseline pre-immunotherapy period of 2011–2012, 3,087 patients were newly diagnosed with advanced non-squamous cell carcinoma; this population served as a basis for survival comparisons between study periods. During 2015–2016 (pre-immunotherapy era), 2017–2018 (post-immunotherapy, availability of second-line SACT), and 2019 (post-immunotherapy, availability of first-line SACT, no pandemic), 3,114, 3,251, and 1,620 patients were newly diagnosed with non-squamous cell carcinoma, respectively. We conducted separate survival analyses among patients diagnosed in 2020 and 2021. In these patient populations, follow-up times were not sufficient for the calculation of 3-year survival rates, nevertheless, they were included in Cox regression analyses.

Among patients diagnosed with late-stage non-squamous cell carcinoma in 2011–2012, 1-, 2- and 3-years survival rates were 50.9% (95% CI: 49.1%–52.6%), 24.8% (95% CI: 23.3%–26.3%) and 14.5% (95% CI: 13.2%–15.7%), respectively (**Figure 3A**). There were no significant changes in survival rates between patients diagnosed in 2011–2012 and 2015–2016. However, patients diagnosed in 2017–2018 had 1-, 2-, and 3-year survival rates of 55.7% (95% CI: 54.0%–57.4%), 32.2% (95% CI: 30.6%–33.8%) and 22.5% (95% CI:21.1%–24.0%), respectively, corresponding to significant, 4.8% (95% CI: 2.4%–4.1%), 7.4% (95% CI: 5.1%–5.4%) and 8.1% (95% CI: 6.2%–6.1%) increases compared to 2011–2012. Patients diagnosed in 2019 who received first-line SACT had a 3-year survival rate of 28.7% (95% CI: 26.4%–30.9%), almost double the survival of those diagnosed in 2011–2012 (14.2% increase; 95% CI: 11.6%–10.3%). The highest 3-year survival rate was found in the age group of 20–59 years (30.6% 95% CI: 27.3%–33.9%). Estimated 1-year survival rates for patients diagnosed during the Covid-19 pandemic in 2020 and 2021 were 57.5% (95% CI: 55.0%–60.0%) and 57.5% (95% CI: 54.8%–60.2%), respectively.

Among patients with squamous cell carcinoma, 3-year survival rates increased from 13.4% (95% CI: 11.8%–15.0%) to 22.3% (95% CI: 19.4%–25.2%) between the 2011-2012 and 2019 diagnostic periods, corresponding to an absolute increase of 8.9% (95% CI: 5.6%–12.2%). One-year survival rates for the 2020 and 2021 diagnostic periods were 56.2% (95% CI: 52.6%–59.8%) and 60.1% (95% CI: 56.3%–64.0%), respectively. 3-year survival rates of small cell lung cancer patients are detailed in **Supplementary Figure 1**.

### Mortality risk in different study periods compared to baseline

**Figure 4:**
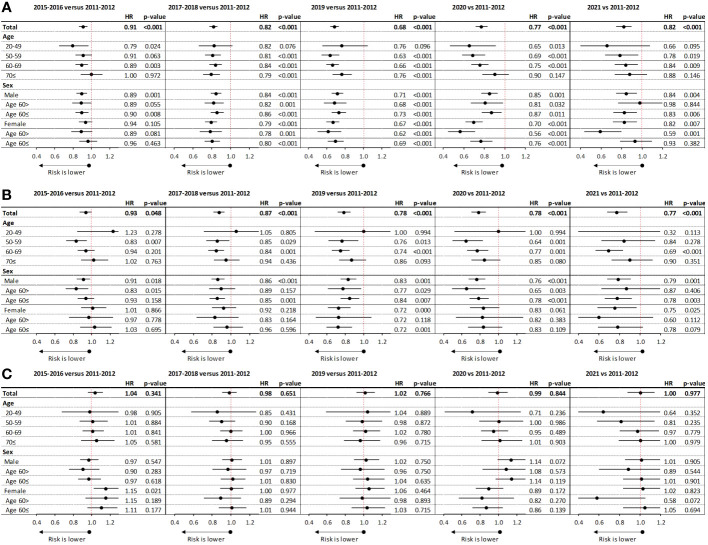
Change in mortality risk compared to the baseline diagnostic period of 2011–2012 among patients with advanced non-squamous cell carcinoma **(A)**, squamous cell carcinoma **(B)**, and small cell LC **(C)** receiving SACT. LC, lung cancer; SACT, systemic anticancer treatment.

Compared to the baseline diagnostic period of 2011–2012, the mortality risk of patients diagnosed with non-squamous cell carcinoma was significantly, 9.00% lower in 2015–2016 (HR: 0.91; p<0.001), 18.2% lower in 2017–2018 (HR: 0.82, p<0.001), and 31.8% lower in 2019 (HR: 0.68; p<0.001) (**Figure 4A**). Improvements were consistent in all age groups and in both sexes in the 2017–2018 and 2019 diagnostic periods. During the first years of the Covid-19 pandemic (2020 and 2021), the reduction in mortality risk remained significant compared to baseline (HR: 0.77 and HR: 0.82, respectively; p<0.001 for both), albeit with less pronounced reductions in older age groups and in male patients.

Patients with squamous cell carcinoma also experienced significant reductions in mortality risk between 2011–2012 and 2015–2016 (HR: 0.93; 95% CI: 0.87–1.00; p=0.048), with more pronounced improvements observed in younger age cohorts and female patients. Patients diagnosed in 2017–2018 and 2019 also had significantly, 12.9% and 22% lower mortality risk compared to those diagnosed in 2011–2012, respectively (HR: 0.87; 95% CI: 0.81–0.93; p<0.001 and HR: 0.78; 95% CI: 0.71–0.86; p<0.001, respectively) (**Figure 4B**). In 2020 and 2021, improvements remained consistent compared to the baseline diagnostic period (HR: 0.78 and HR: 0.77, respectively; p<0.001 for both). There were no significant reductions in the mortality risk of patients with small cell carcinoma in any of the examined diagnostic periods compared to 2011–2012 (**Figure 4C**).

### Treatment patterns

**Figure 5:**
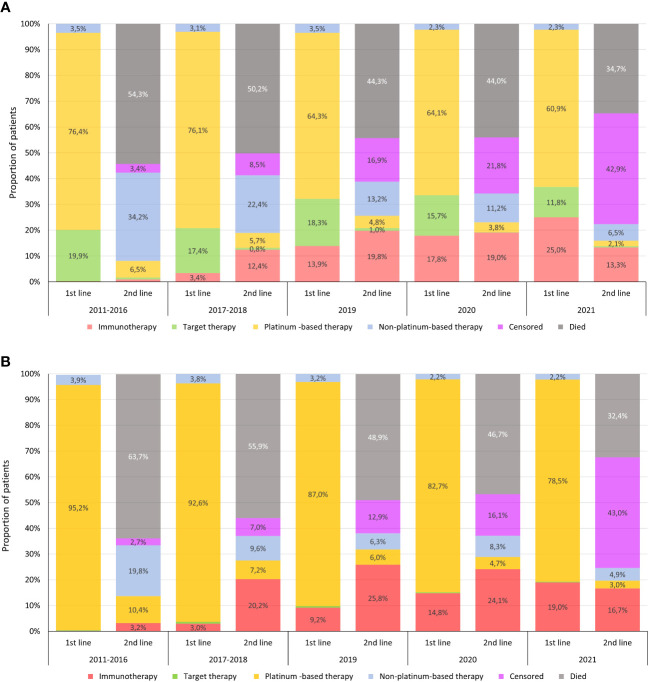
Treatment patterns of non-squamous cell carcinoma **(A)** and squamous cell carcinoma **(B)** patients in first- and second-line regimens. Censored in the columns of second-line therapy means that the patient was still receiving first-line treatment at the end of the respective period.

The majority of non-squamous cell carcinoma patients diagnosed during the 2011–2016 period received platinum-based chemotherapy as first-line treatment (76.4%); 19.9% of them received targeted therapy with tyrosine kinase inhibitors (TKI), of whom 34.2% continued with second-line non-platinum-based therapy (**Figure 5A**). Among patients diagnosed in 2017–2018, first-line treatment patterns were similar to the 2015–2016 period, however, 12.45% of all non-squamous cell carcinoma patients received immunotherapy in second line. In patients diagnosed in 2019, 64.3% received a platinum-based regimen, 18.3% received TKI, and 13.9% received immunotherapy as first-line treatment, and 19.8% of all patients received immunotherapy in second line. The proportion of first-line immunotherapy increased to 17.8% and 25% by 2020 and 2021, respectively.

In the squamous cell carcinoma population, platinum-based therapy was dominant in first line during the 2011–2016 pre-immunotherapy period (95.2%), and most patients received non-platinum-based regimens in second line (**Figure 5B**). For patients diagnosed in 2017–2018, immunotherapy became the leading treatment choice in second line, with 20.2% of all squamous cell carcinoma patients receiving second-line immunotherapy, more than half of all patients in second line. The proportion of first-line immunotherapy was 9.2% in patients diagnosed in 2019, and it was the dominant second-line choice in this diagnostic period. The proportion of patients receiving first-line immunotherapy further increased to 14.8% and 19.0% in patients diagnosed in 2020 and 2021, respectively. For adenocarcinoma patients, the aggregate percentage of individuals who received I-O treatment across various treatment lines exhibited the following trends: 25.5% in 2017–18, 37.1% in 2019, 38.9% and 35.7% in 2020 and 2021. Corresponding percentages in the squamous cell carcinoma population over the same time frame were 20.9%, 37.1%, 38.4% and 38.3%, respectively.

We found no significant changes in treatment patterns among patients with small cell carcinoma diagnosed in different study periods.

**Figure 6:**
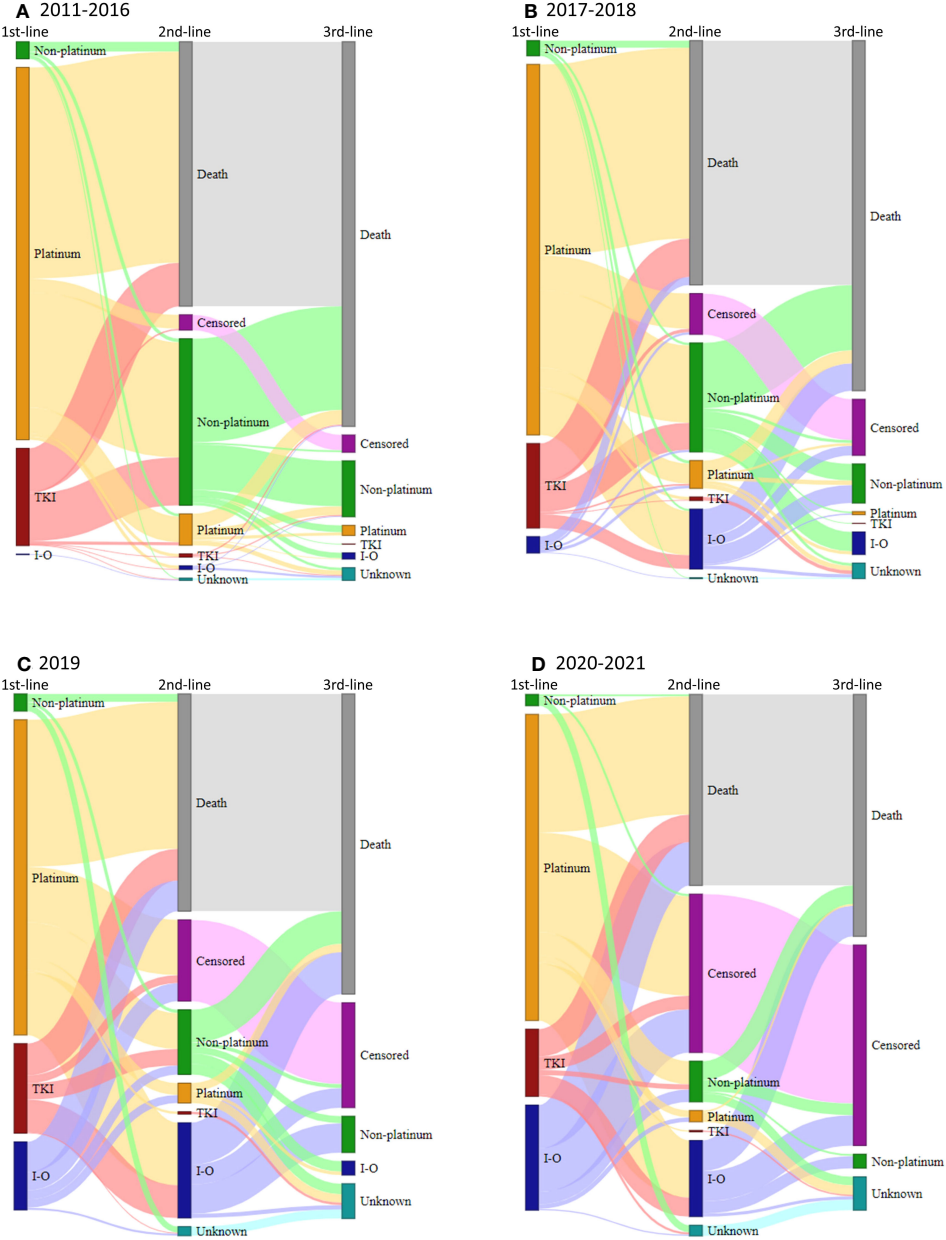
Treatment sequencing for patients who received first-line treatment for advanced LC with non-squamous cell carcinoma histology during the pre-I-O **(A)**, I-O second line **(B)**, I-O first line **(C)**, and Covid-19 pandemic periods **(D)**, respectively, shown on Sankey diagrams. Platinum, platinum-based chemotherapy; I-O, immunotherapy; non-platinum, non-platinum-based chemotherapy; TKI, tyrosine kinase inhibitor.

**Figure 6** shows treatment sequencing in the non-squamous cell carcinoma patient population during different diagnostic periods. A significant proportion of patients died after each treatment line, therefore, only few patients received third-line treatment. Patients who were still alive at the end of the follow-up time (30 June 2022) without any change of therapy from the previous line were censored. The proportion of censored patients increases towards the end of the study period due to the different follow-up times of patients diagnosed in different study periods. Treatment sequencing in the squamous cell carcinoma patient population is detailed in **Supplementary Figure 2**.

## Discussion

This nationwide, retrospective analysis provides overall survival data for patients with lung cancer who received systemic anti-cancer therapy during the past decade in Hungary based on a comprehensive data source. To our knowledge, our study is the first to report changes in LC survival during the Covid-19 pandemic as well as among the first to provide survival estimates for patients receiving SACT after the introduction of immunotherapy for LC in Hungary.

The main findings of our large-scale analysis can be summarized as follows:

1. The mortality risk of patients with advanced LC who received SACT significantly decreased by 32% in patients with non-squamous cell carcinoma and by 22% in patients with squamous cell carcinoma over the past decade. The most significant change could potentially be attributed to the introduction of immunotherapy, especially the availability of first-line immunotherapy as well as to the development of TKI treatments. We did not find any similar improvements among patients with small cell LC during the study period.
2. 3-year survival rates almost doubled among patients with non-squamous cell carcinoma (28.7% vs. 14.5%) and significantly improved in patients with squamous cell carcinoma (22.3% vs. 13.4%) between 2011–2012 and 2019.
3. The improvement in OS was consistent across all age groups and was slightly more pronounced in females in the NSCLC population.
4. The OS improvement remained significant among patients who were diagnosed with LC during the Covid-19 pandemic, albeit with smaller improvements in older age groups and male patients.

The significant improvement in the survival of advanced LC is associated with the introduction of immunotherapy as a second-line treatment option in 2017 and its subsequent availability in first line starting from 2019. Immunotherapy agents have been shown to provide significant survival benefits for patients with advanced LC who had had a poor prognosis before the immunotherapy era (10–12, 14).

The survival advantage provided by immunotherapy seen in clinical studies has been confirmed by several real-world analyses (29, 30). A study from Slovenia reported 12-month OS rates of 64% and 35% among patients with metastatic NSCLC receiving immunotherapy in first and second line, respectively (29). A Japanese study found a 12-month OS rate of 58.3% in patients with advanced or recurrent NSCLC receiving first-line pembrolizumab treatment (30). A study from the U.S. reported an estimated 12-month OS of 59.5% among patients with metastatic non-squamous cell NSCLC receiving immunotherapy in combination with chemotherapy (31). On the other hand, a study conducted in the Netherlands found lower OS with first- and second-line immunotherapy compared to clinical study results, however, outcomes for chemotherapy and targeted therapy were still somewhat poorer than expected based on their respective RCTs (19).

In our study, patients with non-squamous cell carcinoma diagnosed in 2019 had significant lower mortality risk compared to those diagnosed in 2011–2012. Survival rates at 12 and 24 months were notably higher, and 3-year survival rates showed substantial improvements. Similarly, patients with squamous cell carcinoma also experienced a significant reduction in mortality risk and improved survival rates during this period.

These improvements coincided with significant changes in first- and second-line treatment patterns, with immunotherapy gaining more use in both patient populations. Our results are in line with the I-O-optimize studies including the recently published population-based real-world analysis from Canada which also compared survival rates in the pre- and post-immunotherapy era in patients with advanced NSCLC (7). In this study, the 2-year OS of patients with non-squamous cell carcinoma was 26% in the pre-immunotherapy era (January 2010 to March 2016) and increased to 34% in the post-immunotherapy era (April 2016 to June 2019), with 15.4% and 38.7% of patients receiving first- and second-line immunotherapy in the post-immunotherapy era, respectively. The studied LC patient population was very similar to the population in our current analysis, since both included patients with late-stage, advanced LC (mostly stage IIIb and IV) receiving only SACT during almost the same study period and with similar developments in treatment patterns and comparable results among patients with squamous cell carcinoma.

In the pre-immunotherapy era, patients with non-squamous cell carcinoma and squamous cell carcinoma had poor prognosis in our study. Our results from this period are comparable to results from the Portuguese I-O-optimize study which reported 2-year survival rates of 17% and 11% among patients with stage IIIb and IV non-squamous cell LC and squamous cell carcinoma, respectively (6), as well as to results from the U.K. showing 3-year survival rates of 8–19% and 6–12% in the same patient populations, respectively (32).

It is important to emphasize that TKI therapies became available for NSCLC patients with *EGFR* or *ALK* mutation during this period in Hungary. First-generation TKIs including erlotinib, gefitinib, and crizotinib became available in 2014, which may have been responsible for at least part of the survival improvement seen in the 2015–2016 period. New generation TKIs such as afatinib for patients with *EGFR* mutation and alectinib for patients with *ALK* mutation were approved in 2016 and 2017, respectively, while osimertinib became available in 2019. Although these first- and new generation TKIs improved the survival for patients with non-squamous cell carcinoma and *EGFR* or *ALK* mutation (33), these patient populations accounted for a low percentage of the overall NSQ group (approx. 10% and 4% based on clinical references). Therefore, the impact of these agents on survival improvement may not have been significant. In addition, our study demonstrated similar survival improvements in the squamous cell carcinoma population, which could not be attributed to TKIs.

The improvement in the survival of Hungarian non-squamous cell carcinoma and squamous cell carcinoma patients was observed during a transition period characterized by significant changes in treatment patterns. In line with international observations, an increasing proportion of Hungarian patients with advanced LC received immunotherapy in first and second line in the post-immunotherapy era, which could largely explain improving outcomes. This is supported by the fact that we found no improvement in survival rates among patients with small cell carcinoma during the study period where immunotherapies were not utilized for treatment. We found consistent improvements in all age groups and slightly more pronounced improvements in female NSCLC patients which may have important implications considering the increasing incidence of LC among women (34).

SARS-CoV-2 infection particularly impacts the respiratory system and may be associated with severe pulmonary complications. The higher severity of SARS-CoV-2 infection among patients with LC is well-documented and may be explained by the pathophysiological, clinical, and treatment-related factors of the underlying condition as well as the smoking-related damage often observed in LC patients and the increased incidence of cardiovascular and respiratory comorbidities (35). Therefore, we might expect an even larger decrease in LC survival during the pandemic years, than in other cancer types (24, 36). Furthermore, certain waves of the Covid-19 pandemic were putting a high burden on healthcare systems, which may have resulted in delays in LC diagnoses, leading to more advanced stages and worse general condition at the time of treatment initiation. Accordingly, a nationwide, population-based modeling study from the U.K. examined the impact of delays in diagnosis on cancer survival and estimated 1,235–1,372 additional deaths within 5 years as well as a 4.8–5.3% decrease in 5-year survival among patients with LC (37). Several studies have examined the impact of the Covid-19 pandemic on LC survival, albeit not specifically in patients with late-stage disease. During the first wave of the pandemic in 2020, a Spanish study reported a higher mortality risk from SARS-CoV-2 infection among elderly LC patients compared to the young, and a higher fatality rate of SARS-CoV-2 infection in patients with LC compared to the non-LC population (25). In addition, a recently published, comprehensive analysis revealed an odds ratio of 4.67 for Covid-19 related mortality among LC patients compared to non-cancer patients, and a 9 times higher risk of death among LC patients with SARS-CoV-2 infection, than non-infected LC patients (38). However, this analysis did not find higher all-cause mortality in the LC patient population during the Covid-19 pandemic compared to the pre-pandemic era.

In our study, we were also expecting to detect a negative impact of the Covid-19 pandemic on the survival of our late-stage NSCLC patient population receiving SACT treatment. However, during the years of the pandemic, the proportion of patients receiving first-line and overall immunotherapy both increased, which also had an impact on the survival of this patient population. In patients with squamous cell carcinoma, survival improvements observed during the pre-pandemic era with the introduction of immunotherapy were maintained during the pandemic years. Improvements were generally more age-dependent and were smaller in male patients and older age groups, potentially due to the higher prevalence of Covid-19 related risk factors in these subgroups. In summary, we observed a much less severe impact of the Covid-19 pandemic on the survival outcomes of patients with advanced NSCLC than expected based on the available knowledge and evidence. This may be explained by the fact that among patients diagnosed during the pandemic years, a significantly higher proportion received first-line immunotherapy compared to those diagnosed before the pandemic.

Our study has certain strengths and limitations. The high number of patients diagnosed with advanced LC, the thorough data cleaning process, the 11-year-long follow-up period, and the nationwide nature of the NHIF database all provide a solid basis for drawing conclusions from our analysis. However, the applied eligibility criteria may have led to the exclusion of patients who had cancer types other than LC, although our estimates suggests that this patient population is negligible. Information on the stage of LC, ECOG PS status, and laboratory test results were not available in the NHIF database, therefore, we were not able to provide specific survival data based on these characteristics. Furthermore, we did not estimate net survival and were not able to perform age-related survival analysis adjusted to the survival rates of the general population or examine the net impact of the Covid-19 pandemic. Nevertheless, considering the much higher mortality of LC patients compared to the general population and the fact that a large proportion of this mortality is caused by LC itself, this limitation is not likely to have a relevant effect on the results and interpretation. Of note, our study was not meant to compare the effectiveness of immunotherapy and traditional chemotherapy, or first- and second-line immunotherapy. A significant proportion of patients diagnosed with LC in the 2017–2021 still received non-immunotherapy treatment, and some patients diagnosed towards the end of the 2011–2016 period may have received immunotherapy in second or further lines. Therefore, we may have underestimated the impact of immunotherapy on survival outcomes, or overestimated survival probability if considering a truly immunotherapy agnostic population in the late pre-immunotherapy era. Finally, the cut-off date for our analysis was 30 June 2022, 2.5 years into the Covid-19 pandemic. The impact of the pandemic could be detected 3–4 years after diagnosis in patients who were diagnosed in 2017–2018, 2 years post-diagnosis in those diagnosed in 2019, but right in the first year among those who were diagnosed in 2020 and 2021. This may have also led to the underestimation of survival during the post-immunotherapy era relative to in the absence of Covid-19, even at different periods of the survival probability curve. It is important to note too that IO therapies were primarily approved for use in metastatic SQ and NSQ patient populations during later time periods. However, it should be emphasized that the cohort as previously defined may also encompass a smaller proportion of locally advanced stage III LC patients. Consequently, the percentage of patients receiving IO treatment may seem lower due to the inclusion of the stage III LC population, where it was not a recommended treatment option. In addition, as regarding the stage III, inoperable lung cancer population, durvalumab, which effectiveness was proven in PACIFIC clinical study (37), was also became available after the 2018 EMEA approval, however, it was not widely reimbursed in Hungary until 2022, hence, could have limited impact on the survival improvement we found in our analysis of late stage SQ and NSQ population. We would like to emphasize that in accordance with our predefined inclusion criteria, survival analyses were only performed in a subset of lung cancer patients diagnosed between 2011 and 2021 who exclusively underwent systemic anticancer treatment (SACT) concurrent with morphology-based diagnoses. Consequently, caution should be exercised when comparing our findings with results from other studies, given the distinctive patient cohort and stringent criteria applied in our analysis. Lung cancer patients who underwent radiotherapy could be incorporated into our study cohorts, as we encountered challenges in precisely discerning the utilization of this therapeutic modality. Consequently, individuals in the earlier phases of the disease who received curative radiotherapy doses may be encompassed within the examined population of lung cancer patients.

## Conclusions

Our nationwide study is among the first to provide short- and mid-term survival data of patients with advanced LC comparing the pre- and post-immunotherapy era in Hungary. We found significant improvements in survival outcomes after the introduction of immunotherapy despite the increase of older age patients during the observation period. The mortality risk of patients with advanced NSCLC receiving SACT decreased by 22–32% over the past decade, and 3-year survival rates were almost twice as high in patients diagnosed in the post-immunotherapy era as in those diagnosed before the availability of immunotherapy agents. 1 Year survival improvements were largely maintained during the Covid-19 pandemic, albeit with less pronounced improvements in male patients and in older age cohorts.

## Data availability statement

The raw data supporting the conclusions of this article will be made available by the authors, without undue reservation.

## Ethics statement

Ethics approval was obtained from the Central Ethical Committee of Hungary (IV/3940- 3 /2021/EKU). The study is based on anonymized data collected for financial purposes by the NHIF of Hungary, thus it does not include images or any other personal data that may be used to identify any person.

## Author contributions

GG: Conceptualization, Methodology, Writing - Original Draft, LT: Supervision, Writing - Review, KB: Conceptualization, Validation, Writing - Review, JM: Supervision, ZKis: Investigation, Conceptualization, Methodology, Writing - Original Draft, Visualization, ZP: Conceptualization, VS, ZP-S, EC, AK-F, ZKir, ZS: Conceptualization, Validation of data, Review VM: Supervision, Validation of data, Review, EK: Validation, KKo, KKn, TS, AB, GH: Methodology, Validation of data, ZV: Methodology, Supervision, GR: Data Curation, ZA-T: Data Curation, Statistical calculations, Visualization, ZB: Writing - Review and Editing. All authors contributed to the article and approved the submitted version.

## Supplementary material

Supplementary Table 1 Patient characteristics of the overall Hungarian LC population and proportion of patients who received SACT. LC: lung cancer; SACT: systemic anticancer therapy.

Supplementary Figure 1 Estimated 3-year overall survival of patients with small cell carcinoma who were diagnosed between 2011–2012, 2015–2016, 2017–2018, and 2019 and received SACT. LC: lung cancer; SACT: systemic anticancer therapy.

Supplementary Figure 2 Treatment sequencing for patients who received first-line treatment for advanced LC with squamous cell carcinoma histology during the pre-IO **(A)**, I-O second line **(B)**, I-O first line **(C)** and Covid-19 pandemic periods **(D)**, respectively, shown on Sankey diagrams. Platinum: platinum-based chemotherapy; I-O: immunotherapy; non-platinum: non-platinum-based chemotherapy; TKI: tyrosine kinase inhibitor.
