# Supplementary figures and images for: Significant changes in advanced lung cancer survival during the past decade in Hungary: impact of modern immunotherapy and the COVID-19 pandemic
Source: Front Oncol. 2023 Oct 4;13:1207295. doi: 10.3389/fonc.2023.1207295 (PMC10584310; doi:10.3389/fonc.2023.1207295)

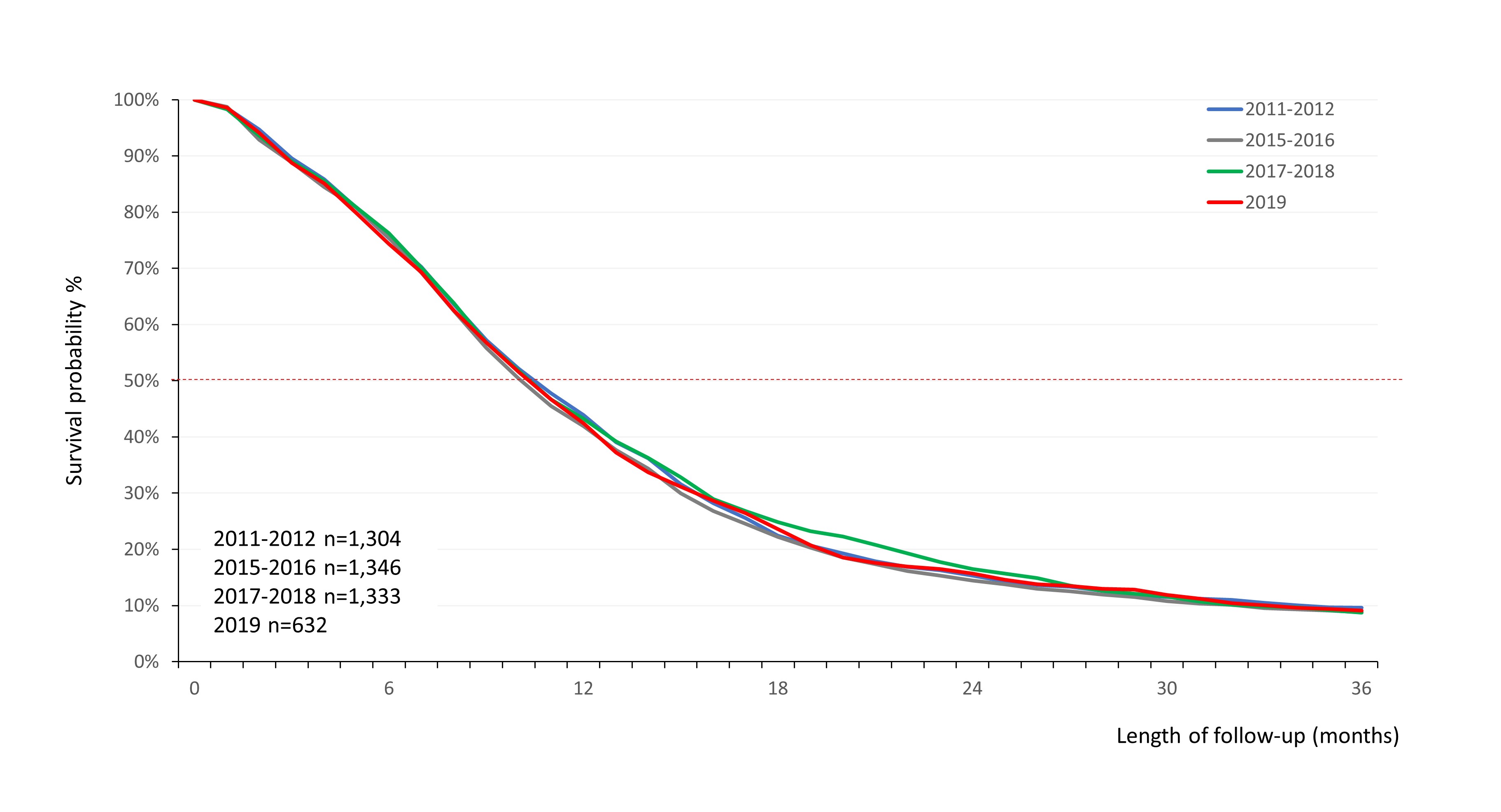

Supplement: Supplementary Figure 1 — Estimated 3-year overall survival of patients with small cell carcinoma who were diagnosed between 2011–2012, 2015–2016, 2017–2018, and 2019 and received SACT. LC: lung cancer; SACT: systemic anticancer therapy. [file Image_1.jpg]

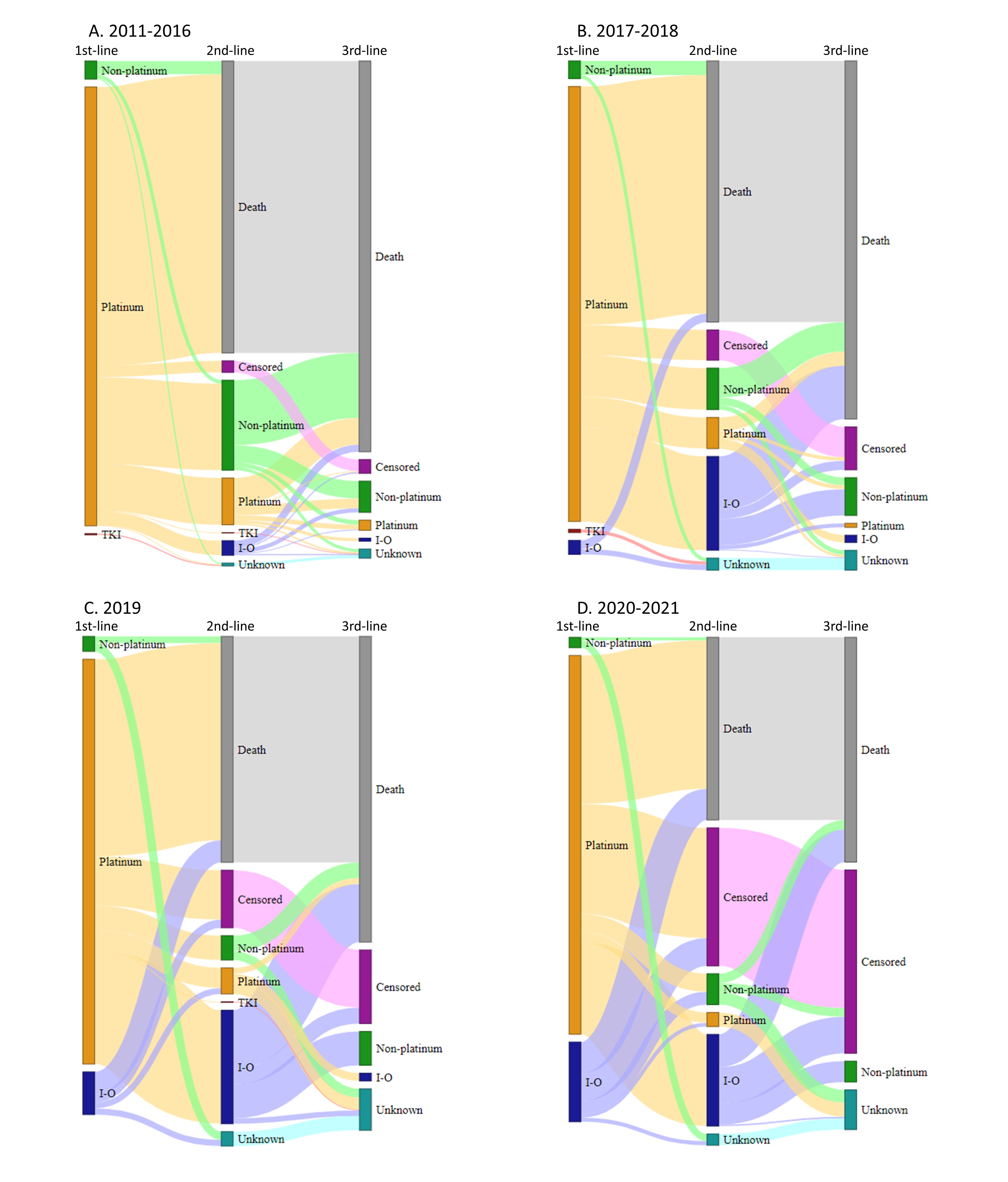

Supplement: Supplementary Figure 2 — Treatment sequencing for patients who received first-line treatment for advanced LC with squamous cell carcinoma histology during the pre-IO (A), I-O second line (B), I-O first line (C) and Covid-19 pandemic periods (D), respectively, shown on Sankey diagrams. Platinum: platinum-based chemotherapy; I-O: immunotherapy; non-platinum: non-platinum-based chemotherapy; TKI: tyrosine kinase inhibitor. [file Image_2.jpg]
